# Would the Dog Be a Person's Child or Best Friend? Revisiting the Dog-Tutor Attachment

**DOI:** 10.3389/fpsyg.2020.576713

**Published:** 2020-10-23

**Authors:** Carine Savalli, Chiara Mariti

**Affiliations:** ^1^Department of Public Politics and Public Health, Federal University of São Paulo, Santos, Brazil; ^2^Department of Veterinary Sciences, University of Pisa, Pisa, Italy

**Keywords:** attachment, bond, caregiver, dog, friendship, tutor

Among all relationships that a human life comprises, there is often the development of interspecific relationships, especially with dogs (Julius et al., 2013). Dogs can cooperate in various scenarios, as they can guide blind people, herd sheep, rescue people, work in animal-assisted therapy, among other activities (Serpell, 2017); and beyond all operational interactions, most tutors and dogs become attached to each other (we are using the term tutor as a synonym of guardian, the one who takes care of the dog). What do people search for in an affectionate relationship with dogs? Are people searching for a new experience of caring for someone who depends on them for basic needs? For an emotional support in difficult times? For a long-term and consistent relationship, a strong connection, a mutually enjoyable contact? In other words, are people searching for a child, for a best friend or both? And how does it work from the dog's perspective? The Bowlby's theory (Bowlby, 1969) focused on child-caregiver attachment is being used to explain dog-tutor attachment. However, we argue that this approach should be integrated with the human friendship attachment theory and the intraspecific dog attachment. Therefore, it is important to revisit the approach to the dog-tutor attachment.

## Child-Caregiver Attachment

The attachment bond encompasses behavioral strategies used by individuals to maximize their survival, by balancing two motivational processes: the need for protection from threats and the drive to explore the environment. A dynamic equilibrium of these two motivational processes is important for the child development (Cassidy, 2016). The attachment figure is the individual who offers comfort in stressful situations (*safe haven effect*) and the security to explore the surroundings (*secure base effect*) (Bowlby, 1969; Ainsworth et al., 1978).

In the child-caregiver attachment, two behavioral systems, namely the attachment system and the caregiving system (Julius et al., 2013), are combined to increase the chances of survival of the offspring. The attachment system is activated in children by emotional stress, triggered by internal or external stimuli, and it includes a set of behaviors used to reestablish the proximity with the caregiver such as calling, crying, etc. The caregiving system is activated in the caregiver by the perception of danger or by the child showing attachment behaviors. Julius et al. (2013) emphasizes that, when child and caregiver interact in synchrony and work together to maintain proximity, both systems are successfully deactivated by physical contact, such as the skin-to-skin contact, which leads to positive feelings and well-being (George and Solomon, 2016). The seek for proximity in non-threatening situations can also occur, and it strengthens the child-caregiver bond (Julius et al., 2013). The caregiver also plays other roles in this relationship, such as educator and a playmate (Cassidy, 2016).

Ainsworth et al. (1978), using the well-known Stranger Situation Test paradigm (ASST), described three styles of child attachment, largely influenced by the caregiver behavior: secure, insecure avoidant, and insecure ambivalent; a fourth style, called disorganized, was introduced by Main and Solomon (1986). According to Cassidy's (2016) deep examination of Bowlby's theory, the systems involved in the child-caregiver attachment also encompass cognitive components such as memory, selective attention, and discriminant learning, among others. Repeated cognitive and affective experiences with the attachment figure form the so-called *internal working models* (Bowlby, 1969) that can influence the way individuals will form future relationships.

## Adult and Friendship Attachment

Hazan and Shaver (1987) stated that, as children grow up, the attachment system does not become inactive but it is, instead, co-opted and influences the development of new bonding in adulthood. During growth, people gradually shift attachment functions from parents to peers, such as a friend or romantic partner (Fraley, 2019). In these bi-directional relationships, each person can interchangeably play the care-seeking and the caregiving roles, depending on specific situations and individual needs.

Although early caregiving experiences continue to influence the attachment orientation in adulthood (Hazan and Shaver, 1987; Chopik et al., 2014), Fraley (2019) argues that this influence can be weaker than previously thought. When two adult individuals develop an affectionate attachment, both can offer and receive support in difficult moments, each one bearing their own earlier experiences, resulting in a dynamic process of adaptation to one another. This plasticity is important for the establishment and maintainance of new relationships (Fraley, 2019), but the comprehension of how attachment patterns change during lifetime remains a challenge.

Adulthood bonding in humans is not a matter of life or death, as it is in childhood (Fraley, 2019). It seems to be driven less by biological needs and more by interpersonal needs. A friendship arises from long-term relationships that present consistency, connectedness, good communication, seeking for, and offering support to each other with high levels of trust, self-disclosure, hope, and relationship satisfaction (Welch and Houser, 2010). Friends also engage in a mutual enjoyable physical contact (Feeney and Woodhouse, 2016; Zeifman and Hazan, 2016), although to a lesser degree than in the child-caregiver dyad. Berndt (2002) noted that a high quality of friendship is characterized by high level of positive features such as pro-social behaviors and is predictive of subjective well-being (Chopik, 2017). Although a friendship usually does not cause separation distress, most theorists describe it as an attachment bond.

Seyfarth and Cheney (2012) used the term friendship to describe enduring social bonds observed in many group-living mammals, suggesting that friendship improves survival, and reproductive fitness. According to them, friendship involves cooperative interactions that can be widely separated in time, depending on memory, and emotions associated with past interactions. Intraspecific friendship is more common in individuals that are genetically related, closer in age and rank; however, it is observed between unrelated individuals (Seyfarth and Cheney, 2012).

## Dog-Dog Attachment

The study of attachment bonds in dogs has focused on their relationship with humans. The presence of an intraspecific attachment bond has instead received scant attention. Studies on separation from conspecifics (Pettijohn et al., 1977; Tuber et al., 1996; Walker et al., 2014) seem to point to a difference in the nature of the social relationships dogs establish with humans and those they establish with conspecifics. Recent studies have also highlighted similarities in the relationship (not attachment) established with humans and with other dogs (Cimarelli et al., 2019).

As for intraspecific attachment, preliminary data suggest that an attachment behavioral system exists in the puppy-mother relationship (Prato-Previde et al., 2009). Although separation stress was observed in an intraspecific version of the ASST (Mariti et al., 2018), Mariti et al. (2014) did not find evidence of an attachment system in intraspecific relationships between adult dogs. In case puppies and mother keep living together in adulthood, the bond between them presents some characteristics of an attachment, more than the bond between two unrelated, co-habitant adult dogs (Mariti et al., 2017).

The use of ASST with couples of co-habitant adult dogs showed that the presence of a human stranger had a stronger ameliorative effect when compared to the presence of an older female dog living in the same household. Nonetheless, the ameliorative effect was almost identical when the stranger was compared to the canine mother (Mariti et al., 2017). The bond between adult dogs does not seem to fit all the characteristics of an attachment bond as intended in a child-caregiver or in a dog-human bond (Mariti et al., 2013). However, results should not be regarded as conclusive, considering the small number of studies on this topic and the peculiar appeal that human beings have to dogs. Such bond might be better investigated using different tools.

It must also be noticed that many factors may impact the kind of relationship dogs establish with conspecifics and the behavior dogs display in the ASST test. For instance, early weaning (Mogi et al., 2011), early separation from littermates (Pierantoni et al., 2011), the amount of maternal care received (Guardini et al., 2017), as well as disruption of the bond with tutors (Prato-Previde and Valsecchi, 2007), are all factors known to affect the development of dogs' social and emotional behaviors. At the same time, the age of the dog might influence the display of attachment-related behaviors both in intraspecific (Carlone et al., 2014) and interspecific tests (Mongillo et al., 2013).

## Dog-Tutor Attachment: A Child-Caregiver or A Friendship Attachment?

Dog–human dyads can establish many different kinds of relationship and bonding (Payne et al., 2015); however, when specifically studying attachment bonds, authors refer to the child-caregiver one (Rehn and Keeling, 2016).

If we compare the dog-tutor bond to the child-caregiver attachment, what would be the role, and the weight, of the attachment system, and caregiving system that the tutor and the dog carry in this relationship? Many questions arise at this point and not all can be easily answered.

The child-caregiver approach explains a good part of the dog-tutor relationship. Most decisions in the dog's life are made by the tutor, who plays the role of caregiver and provider of the dog's needs, including security. The ASST adapted to study the bond developed by the dog toward the tutor has been widely used and has repeatedly shown that dogs behave similarly to children in a stressful situation, seeking for the proximity of their tutors, preferring them to an unfamiliar person and exploring their surroundings more when tutors are present (e.g., see Topál et al., 1998; Palmer and Custance, 2008; Mongillo et al., 2013; Mariti et al., 2018; Carlone et al., 2019). Both the secure base (Mariti et al., 2013) and the safe haven effect (Gácsi et al., 2013) have been observed in the dog-tutor bond. Preliminary data also suggest that dogs tested in the ASST with their tutors present similar attachment styles as children (Solomon et al., 2019).

However, the dog-tutor relationship is a more complex phenomenon. For almost their lifetime together tutor and dog are adult individuals, from different species. The well-distinguished roles of the child and caregiver are not fixed in the dog-tutor attachment. The relationship is less asymmetrical and more reciprocal than the child-caregiver bond. Dogs can also represent an attachment figure for people. Separation from the dog can trigger anxiety and anguish in the tutor (Zilcha-Mano et al., 2011), while the close presence of the dog makes the tutor more confident in thinking about future goals and how to accomplish them (Zilcha-Mano et al., 2012). Sometimes the dog represents comfort and emotional support to the tutor in moments of distress (Zasloff, 1996; Dwyer et al., 2006). In this sense dogs also play a role like a secure base and safe haven for the tutor.

We can hypothesize that dogs, as humans, can carry both attachment and caregiving systems into their adult lifetime. Based on the literature, we suggest that dogs may have: an attachment system, activated by emotional stressful situations and deactivated by the proximity/contact with their tutor; and a caregiving system, activated by the dog's perception of distress or danger surrounding the tutor and deactivated by the tutor's signals of recovered well-being. Skills such as emotion recognition (Albuquerque et al., 2016) and empathy (Custance and Mayer, 2012) toward humans have already been recognized in dogs. This empathic ability motivates prosocial and helping behaviors, as demonstrated in studies in which dogs rescued their tutors from a distressful situation (Sanford et al., 2018; Carballo et al., 2020; Van Bourg et al., 2020). These evidences reinforce the plausibility of the hypothesis that dogs can also carry a type of caregiving system, but more studies are needed to better investigate the role of caregiver in dogs.

On one hand, the friendship attachment theory seems to partially explain the dynamic process of adaptation of dog and tutor to one another, combining two strategies of offering and receiving support in difficult moments. From the human's point of view, a relationship with a dog appears to be driven by interpersonal needs, a search for a long-term relationships with consistency, connectedness and closeness (Kurdek, 2009), which also resembles what a friendship offers for people (Welch and Houser, 2010; Chopik, 2017). From the adult dog's perspective, the relationship with a human is not a matter of life or death, stray dogs, for example, survive. Then, dogs also have different motivations than children to develop an attachment to their tutors, and they have a notably appeal for relationships with humans (Lazzaroni et al., 2020).

On the other hand, the child-caregiver attachment theory remains important in explaining the frequent and intense body contact between tutor and dog. The skin-to-skin contact triggers oxytocin release (see Julius et al., 2013, for a deeper discussion), the increase of which has also been demonstrated in affiliative interactions between dogs and humans (Nagasawa et al., 2009, 2015; Handlin et al., 2011, 2012). This important aspect makes the interspecific bond similar to the child-caregiver attachment.

Although along this opinion piece we focused our analyses in the relationship between an adult dog and an adult tutor, it must be noticed that, when they are puppies, dogs have the opportunity to establish a young-caregiver attachment bond, which adds even more complexity to this discussion, since it can involve a mother and/or a human caregiver (Prato-Previde et al., 2009; Mariti et al., 2020).

For social species, natural selection would have favored individuals who are motivated to form long-term bonds, not exclusively with kin (Seyfarth and Cheney, 2012). The dog-tutor attachment represents a strong, long-term bond that goes beyond the species. Whether the dog is the tutor's child or the best friend, or both, this attachment bond may be adaptative for both species and thus requires further research to be better understood.

## Conclusions

Several arguments support that the dog-tutor relationship comprises characteristics of different types of attachment bonds. We suggest that child-caregiver attachment is not enough to characterize this interspecific bond and that a more integrative theory, that combines child-caregiver and friendship attachment should be considered. For example, while investigating dog-tutor attachment, questionnaires could include characteristics usually present in adult friendship; and behavioral tests could include situations aimed at triggering the caregiving system in dogs, to analyze how dogs offer support for their tutors. By suggesting that dog-tutor attachment integrates characteristics of different kinds of attachment bonds, we hope to provide a better picture of a bond that is one of the most important interspecific affectionate relationships for both species, and which appears to be much more complex than previously considered, a complexity that can be attribute to both parties.

## Author Contributions

CS and CM equally contributed to this opinion manuscript. Both authors reviewed and gave final approval for publication.

## Conflict of Interest

The authors declare that the research was conducted in the absence of any commercial or financial relationships that could be construed as a potential conflict of interest.
